# A phase II study of atezolizumab in combination with stereotactic radiation for patients with triple-negative breast cancer and brain metastasis

**DOI:** 10.1007/s10549-026-07932-6

**Published:** 2026-03-07

**Authors:** Antonio Giordano, Noah Graham, Ayal A. Aizer, Nabihah Tayob, Alyssa M. Pereslete, Jonathan D. Schoenfeld, Jose Pablo Leone, Raechel Davis, Timothy K. Erick, Erica L. Mayer, Eric P. Winer, Ian Krop, Sara M. Tolaney, Nancy U. Lin

**Affiliations:** 1 Dana-Farber Cancer Institute, Dana-Farber Cancer Institute, 450 Brookline Avenue, Boston, MA 02215 USA; 2 Harvard Medical School, Boston, MA USA; 3 Brigham and Women’s Hospital, Boston, MA USA; 4 Yale Cancer Center, New Haven, CT USA; 5 Yale School of Medicine, New Haven, CT USA

**Keywords:** Triple-negative breast cancer (TNBC), Brain metastases, Stereotactic radiosurgery (SRS), Atezolizumab

## Abstract

**Purpose:**

Triple-negative breast cancer (TNBC) patients with brain metastases have a poor prognosis and limited treatment options. Preclinical and clinical evidence suggests that radiotherapy may act synergistically with immune checkpoint inhibitors.

**Methods:**

We conducted an open-label, single-arm, phase II study of atezolizumab plus stereotactic radiosurgery (SRS) in metastatic TNBC patients with brain metastases. The primary endpoint was progression-free survival (PFS) according to the Response Assessment in Neuro-Oncology Brain Metastases (RANO-BM) bi-compartmental model. Secondary endpoints included extracranial objective response rate, overall survival (OS), and safety and tolerability. A safety run-in analysis for dose-limiting toxicity (DLT) was performed after the first 6 patients were enrolled and completed the assessment period.

**Results:**

Six patients were enrolled into the safety run-in phase between May 11, 2018 and October 24, 2019. No DLTs were observed, but the study was closed early due to slow accrual. Patients received a median of 2 atezolizumab cycles (range: 2—16), and SRS was administered to all 6 patients. Treatment-related adverse events (TRAEs) occurred in 4 participants (66.7%); all events were grade 2. The median bi-compartmental PFS was 1.3 months (95% confidence interval (CI): 0.95 – NA) and the median OS was 9.7 months (95% CI: 3.6 – NA). The best observed response by RECIST 1.1 criteria was stable disease ≥ 24 weeks in one participant (16.7%).

**Conclusions:**

Concurrent SRS with atezolizumab was feasible in TNBC patients with brain metastases. However, disease outcomes were poor, and the development of effective therapies for these patients remains a significant unmet medical need.

**Clinical Trial Registry Number:**

https://www.clinicaltrials.gov NCT03483012.

Trial Open to Accrual: 05/01/2018.

**Supplementary Information:**

The online version contains supplementary material available at 10.1007/s10549-026-07932-6.

## Introduction

Triple-negative breast cancer (TNBC), which is negative for estrogen receptor, progesterone receptor and human epidermal growth factor receptor 2 (HER2), accounts for roughly 15% of breast cancer cases [1]. Compared to patients with other breast cancer subtypes, patients with early-stage TNBC have a heightened risk of distant recurrence and death within five years of diagnosis [2, 3]. Overall survival (OS) among TNBC patients undergoing systemic therapy for metastatic disease ranges from 13–23 months [4, 5].

The central nervous system (CNS) is a common site of metastasis for TNBC patients. Approximately 25% to 46% of patients with metastatic TNBC develop CNS metastases [2, 6–8], which can occur in the brain parenchyma or along the leptomeninges. Brain metastases are typically treated with surgery and/or radiation therapy, which can consist of whole-brain radiation therapy (WBRT) or stereotactic radiosurgery (SRS) [9]. Despite optimal CNS-directed local therapy, survival outcomes remain poor, with median OS ranging from 3–7 months from the date of diagnosis of brain metastases [10–13].

Preclinical and clinical evidence suggests that radiation therapy (RT) may enhance the efficacy of immune checkpoint inhibitors (ICIs) in solid tumor patients with brain metastases. In mouse models of breast cancer, RT and ICIs act synergistically to upregulate tumor-associated antigen-MHC complexes, enhance antigen cross-presentation, upregulate programmed death ligand 1 (PD-L1) expression, promote the infiltration of antigen-specific T cells, and reduce the accumulation of myeloid-derived suppressor cells [14, 15]. Retrospective studies in melanoma patients with brain metastases have shown that concurrent treatment with SRS and ipilimumab or nivolumab is well-tolerated and associated with improved survival [16–18]. The available data also suggest that concurrent use of ICIs and SRS for brain metastases from non-small cell lung cancer (NSCLC) is tolerable and may be more effective than sequential treatment with radiotherapy and ICI [19–21]. As a result of this synergy, reports of an effect at distant disease sites, known as the *abscopal effect*, have been reported [22]. However, when this study was designed, the combination of SRS with an ICI had not been evaluated prospectively in TNBC patients with brain metastases.

To evaluate the safety and potential synergy between RT and ICI in TNBC, we conducted a phase II study of atezolizumab in combination with SRS in TNBC patients with brain metastases.

## Methods

### Study design and patient population

This was a single-institution, open-label, single-arm, phase II study of atezolizumab with concurrent SRS in patients with metastatic TNBC with brain metastases. Eligible patients had histologically or cytologically confirmed stage IV TNBC, defined as estrogen receptor < 1%, progesterone receptor < 1%, and HER2-negative per American Society of Clinical Oncology/College of American Pathologists (ASCO/CAP) guidelines [23]. Participants had radiologically confirmed brain metastases, consisting of ≤ 5 new or progressive lesions in the brain (each ≤ 3 cm in diameter in any direction) requiring SRS. Patients may have had more than 5 total brain lesions, as long as no more than 5 required SRS treatment. To evaluate for potential abscopal effect, participants also had measurable extracranial disease as defined by RECIST 1.1 criteria [24].

Participants must not have received prior treatment with any anti-PD-1, PD-L1, or PD-L2 agent. There was no limit to the number of prior lines of systemic therapy, and participants who had not received any prior systemic therapy were also eligible. All participants were at least 18 years old with an Eastern Cooperative Oncology Group (ECOG) performance status of ≤ 2 and normal organ and bone marrow function as defined by institutional standards.

Patients with known leptomeningeal or brainstem metastases were ineligible. In addition, participants must not have had CNS complications for which urgent neurosurgical intervention was indicated (such as resection or shunt placement). Treatment with high dose systemic corticosteroids (defined as dexamethasone > 2 mg/day or bioequivalent) within 7 days of initiating therapy was not allowed. Additional exclusion criteria included pregnancy or breastfeeding, known infection with human immunodeficiency virus, hepatitis B virus, or hepatitis C virus, uncontrolled intercurrent illness, or history of different malignancy (except for participants who remained disease-free for at least 3 years or had a low risk of recurrence as deemed by the principal investigator).

### Treatment and procedures

Participants received SRS in combination with atezolizumab 1200 mg IV on day 1 of each 21-day (3-week) cycle. No additional concurrent anti-cancer therapy, including chemotherapy, was allowed. The first dose of atezolizumab was administered 2–7 days before initiating SRS. SRS was typically initiated within 14 days of the planning brain magnetic resonance imaging and delivered framelessly using a linear accelerator with a limited number of isocenters. The standard SRS dose was 20 Gy; however, dose reductions were permitted for larger tumors to limit the volume of normal brain receiving ≥ 12 Gy to ≤ 10 cc, as well as for disease in or near the brainstem or optics. If this constraint could not be met at 16 Gy, treatment was converted for intact tumors to hypofractionated SRT using 30 Gy in five fractions. RT was delivered at Brigham and Women’s Hospital or Dana-Farber Cancer Institute.

Atezolizumab treatment was administered for an indefinite number of cycles until a participant experienced disease progression by Immunotherapy Response Assessment in Neuro-Oncology (iRANO) [25] and/or Immune-Related Response Criteria (irRC) [26], unacceptable adverse event(s), intercurrent illness that precluded further treatment, or any changes to the participant’s condition that rendered them unsuitable to continue receiving atezolizumab in the opinion of the treating investigator.

Participants were required to undergo a research biopsy at baseline (before the initiation of study therapy) and at Cycle 2, Day 1 if extracranial metastases were safely accessible. Participants without biopsy-accessible disease were required to submit an archival primary and/or metastatic specimen. The research biopsies could be waived with principal investigator approval for the first 6 participants enrolled to the safety run-in phase of the study.

### Assessments

Participants removed from study therapy for unacceptable adverse event(s) were followed until resolution or stabilization of the event(s). Participants removed from study therapy for extracranial progression in the setting of intracranial response or stable disease were followed for CNS progression and survival after removal from protocol therapy. Participants removed from protocol therapy for intracranial disease progression were followed until death.

To assess for potential delayed radiation toxicity, a 6-month safety visit was performed. Patients who discontinued protocol therapy before the 6-month SRS treatment visit were evaluated by phone and had records reviewed to achieve complete ascertainment of this endpoint.

Investigators evaluated the participants’ neurological function using the Neurologic Assessment in Neuro-Oncology (NANO) scale [27]. Patient-reported outcomes (PROs) were measured by the M.D. Anderson Symptom Inventory-Brain Tumor (MDASI-BT) assessment [28] and the EQ-5D evaluation [29]. PROs were completed at baseline, on Day 1 of Cycles 3, 5, 9, and once participants were off treatment.

### Statistical considerations

A safety run-in analysis was planned after the first 6 patients were enrolled and completed the assessment period for dose-limiting toxicity (DLT), which was the period from the first dose of atezolizumab until Cycle 3, Day 1. DLT included any of the following events: death; grade ≥ 3 treatment-emergent neurological toxicity; asymptomatic grade 4 hematologic toxicity lasting ≥ 14 days (unless deemed by the investigator to be clinically insignificant); grade ≥ 4 thrombocytopenia (any duration) or grade ≥ 3 thrombocytopenia (if associated with bleeding); grade ≥ 3 febrile neutropenia; grade 3 aspartate aminotransferase (AST) or alanine aminotransferase (ALT) elevation associated with grade 2 bilirubin elevation at least possibly related to study drug (Hy’s Law); grade 3 non-hematologic toxicity that either required medical intervention, led to hospitalization, or persisted for > 7 days (excluding alkaline phosphatase (ALP) ≤ 10 × upper limit of normal [ULN] in a patient with baseline grade ≥ 2 ALP elevation from bone metastasis, or any other laboratory values the investigator deemed clinically insignificant); grade ≥ 3 pneumonitis (any duration); grade ≥ 3 fatigue (> five days); and any other grade ≥ 3 non-laboratory toxicity lasting ≥ three days despite optimal supportive care (excluding alopecia of any grade). If fewer than 3 DLTs were observed within the first 6 patients enrolled, the study would proceed to full accrual. If 3 or more DLTs were observed in the first 6 patients assessed, the study would be closed to further enrollment. With this strategy, there was a 90% probability of continuing enrollment if the true DLT rate was 20%, and a 34% probability of continuing enrollment if the true rate was 50%.

All participants who received at least one dose of study therapy were included in the efficacy analyses. The primary endpoint was bi-compartmental progression-free survival (PFS), defined as time from first dose of atezolizumab (Cycle 1, Day 1) to progression or death from any cause. Progression was defined according to the bi-compartmental model proposed by the Response Assessment in Neuro-Oncology Brain Metastases (RANO-BM) working group, and defined as the first detection of radiologic progression of intracranial disease (per RANO-BM criteria), extracranial disease (per RECIST 1.1 criteria), or both; or unequivocal progression of non-measurable disease in the treating physician’s opinion, with each compartment (CNS and non-CNS) assessed separately [30]. A sample size of 45 participants was chosen to achieve 80% power to detect a difference between a median PFS of 2 months (null hypothesis) [31, 32] and 3.5 months (alternative hypothesis) at a one-sided type I error of 0.05.

Key secondary endpoints included OS, defined as the time from the first dose of atezolizumab to death from any cause; and extracranial objective response rate (abscopal response), defined as an extracranial complete response (CR) or partial response (PR) according to RECIST 1.1 criteria. Because of the strong interest in exploring a potential abscopal effect, evaluable extracranial disease was required. Based on prior studies evaluating ICI in metastatic TNBC (which excluded patients with active CNS metastases) [33, 34], the null hypothesis was that the extra-CNS response rate is 10% or lower. With the sample size of 45 patients required for the primary endpoint, there would be 82% power to reject the null hypothesis if the true extra-CNS response rate is 25%. Based on a high CNS response rate previously reported with SRS alone [35], we did not anticipate a detectable increase in the CNS response rate for SRS given in combination with immune checkpoint therapy.

SRS-treated lesions could be designated as target lesions if they were reproducibly measurable and at least 1 cm in the longest dimension. If a patient entered the study with previously treated and stable CNS lesions, they were not retreated as part of the study. These lesions were designated as non-target lesions for the purpose of this study.

## Results

### Patient and treatment characteristics

A total of 6 patients were enrolled into the safety run-in phase of the study between May 2018 and October 2019 (Supplementary Table 1). All 6 participants were women, with a median age of 46 years (range: 32–69). Most had initially been diagnosed with stage I (16.7%) or II disease (66.7%), and most had a disease-free interval greater than two years (66.7%). All participants had received neoadjuvant or adjuvant anthracycline and taxane chemotherapy, though most had received 0 (33.3%) or 1 (50%) prior line of chemotherapy for metastatic disease. Among the 6 patients, 1 patient was on dexamethasone 2 mg per day (allowed by protocol) (Table 1). Two subjects (cases 3 and 6) had CNS target lesions, and the overall median size at baseline was 10.8 mm.

**Table 1:** Baseline patient, tumor, and treatment characteristics

Characteristic, N (%)	All patients (*N* = 6)
Age at registration, Years	
Median (range)	46 (32—69)
< 50	4 (66.7%)
≥ 50	2 (33.3%)
Sex	
Female	6 (100.0%)
Race	
White	5 (83.3%)
Other	1 (16.7%)
Ethnicity	
Hispanic or Latino	1 (16.7%)
Non-Hispanic	5 (83.3%)
ECOG PS at Baseline	
0	5 (83.3%)
1	1 (16.7%)
Stage at Initial Diagnosis	
I	1 (16.7%)
II	4 (66.7%)
Not IV but otherwise unknown	1 (16.7%)
Disease-free interval^a^	
≤ 2 years	2 (33.3%)
≥ 2 years	4 (66.7%)
Hormone receptor status of primary tumor	
ER-positive/PR-negative	1 (16.7%)
ER- and PR-negative	5 (83.3%)
Hormone receptor status of metastatic tumor	
ER- and PR-negative	5 (83.3%)
Not done	1 (16.7%)
HER2 status of primary tumor (IHC)	
Negative (0,1 +)	4 (66.7%)
Equivocal (2 +)	1 (16.7%)
Not Done	1 (16.7%)
HER2 status of primary tumor (ISH)	
Negative (copy number < 4 and HER2/CEP17 ratio < 2.0)	4 (66.7%)
Not Done	2 (33.3%)
Measurable disease by RECIST 1.1 at baseline	
Yes	6 (100.0%)
Baseline biopsy performed	
No (“PI waived”)	6 (100.0%)
Sites of disease at study entry^b^	
CNS	6 (100.0%)
Lung	3 (50.0%)
Liver	3 (50.0%)
Bone	2 (33.3%)
Breast or chest wall	2 (33.3%)
Other lymph nodes	3 (50.0%)
Hilar, Mediastinal	1 (33.3%)
Retroperitoneal Aortocaval	1 (33.3%)
Mediastinal, Supraclavicular, Hilar, Retroperitoneal	1 (33.3%)
Other	3 (50.0%)
Adrenal	1 (33.3%)
Pleura	2 (66.7%)
Adjuvant or neoadjuvant endocrine therapy	
Yes	3 (50.0%)
No	3 (50.0%)
Adjuvant or neoadjuvant chemotherapy^b^	
Anthracycline	6 (100.0%)
Taxane	6 (100.0%)
Other	2 (33.3%)
Lines of Chemotherapy for Metastasis or Recurrence	
None	2 (33.3%)
1 line	3 (50.0%)
5 lines	1 (16.7%)
Prior chemotherapy for MBC^b^	
Abraxane	1 (16.7%)
Capecitabine	1 (16.7%)
Carboplatin	1 (16.7%)
Eribulin	1 (16.7%)
Gemcitabine plus carboplatin	1 (16.7%)
Etirinotecan Pegol	1 (16.7%)
Pertuzumab/Paclitaxel/Trastuzumab	1 (16.7%)
Vinorelbine	1 (16.7%)
Prior endocrine therapy for contralateral breast cancer or MBC	
No	6 (100.0%)
Use of corticosteroids	
No	5 (83.0%)
Yes	1 (17.0%)
Prior brain surgery	
No	6 (100.0%)
Prior brain radiation^b^	
WBRT	1 (16.7%)
SRS	3 (50.0%)
None	3 (50.0%)

^a^Disease-free interval is defined as the time between the diagnosis of primary breast cancer and the diagnosis of metastatic recurrence

^b^Rows are not mutually exclusive; patients may belong to more than one category

*ECOG PS* Eastern Cooperative Oncology Group Performance Score, *ER* estrogen receptor, *PR* progesterone receptor, *HER2* human epidermal growth factor receptor 2, *IHC* immunohistochemistry, *ISH *in situ hybridization, *PI* principal investigator, *CNS* central nervous system, *MBC* metastatic breast cancer, *WBRT* whole brain radiation therapy, *SRS* stereotactic radiosurgery

DLT were observed in 0/6 participants in the safety run-in. The study was closed to further accrual after enrolling the first 6 participants.

### Treatment details

The median number of atezolizumab cycles administered per patient was 2 (range: 2—16). No patients required dose hold or dose reduction (Supplementary Table 2). SRS was administered to all 6 patients; one patient had one metastasis irradiated (16.7%), 2 patients had 2 metastases irradiated (33.3%), 1 patient had 4 metastases irradiated (16.7%), and 2 patients had 5 metastases irradiated (33.3%). All 6 patients who received SRS received 20 Gy of radiation in a single fraction (Supplementary Table 3). The median time from first atezolizumab infusion to SRS was 5.5 days (mean 5.2, range 1–10 days).

### Safety

TRAEs (all grade 2) occurred in 4 participants (66.7%) (Table 2). Adverse events regardless of attribution to study treatment (or treatment emergent adverse events, TEAEs) occurred in five participants (83.3%), and included one instance each (16.7%) of grade 3 fatigue, ataxia, dyspnea, and pleural effusion (Supplementary Table 4). Neither immune-related adverse events nor radiation necrosis were observed in this study.

**Table 2:** Grade 2 or higher adverse events possibly, probably, or definitely related to treatment^a^

	*N* (%^b^) patients with event
Event	Grade 2
Any Event	4 (66.7%)
Fatigue	2 (33.3%)
Alkaline Phosphatase Increased	1 (16.7%)
Anorexia	1 (16.7%)
Constipation	1 (16.7%)
Headache	1 (16.7%)
Urinary Tract Infection	1 (16.7%)

^a^Maximum grade per patient per adverse event

^b^Denominator is the number of patients that received any treatment

### Efficacy

**Fig.1:**
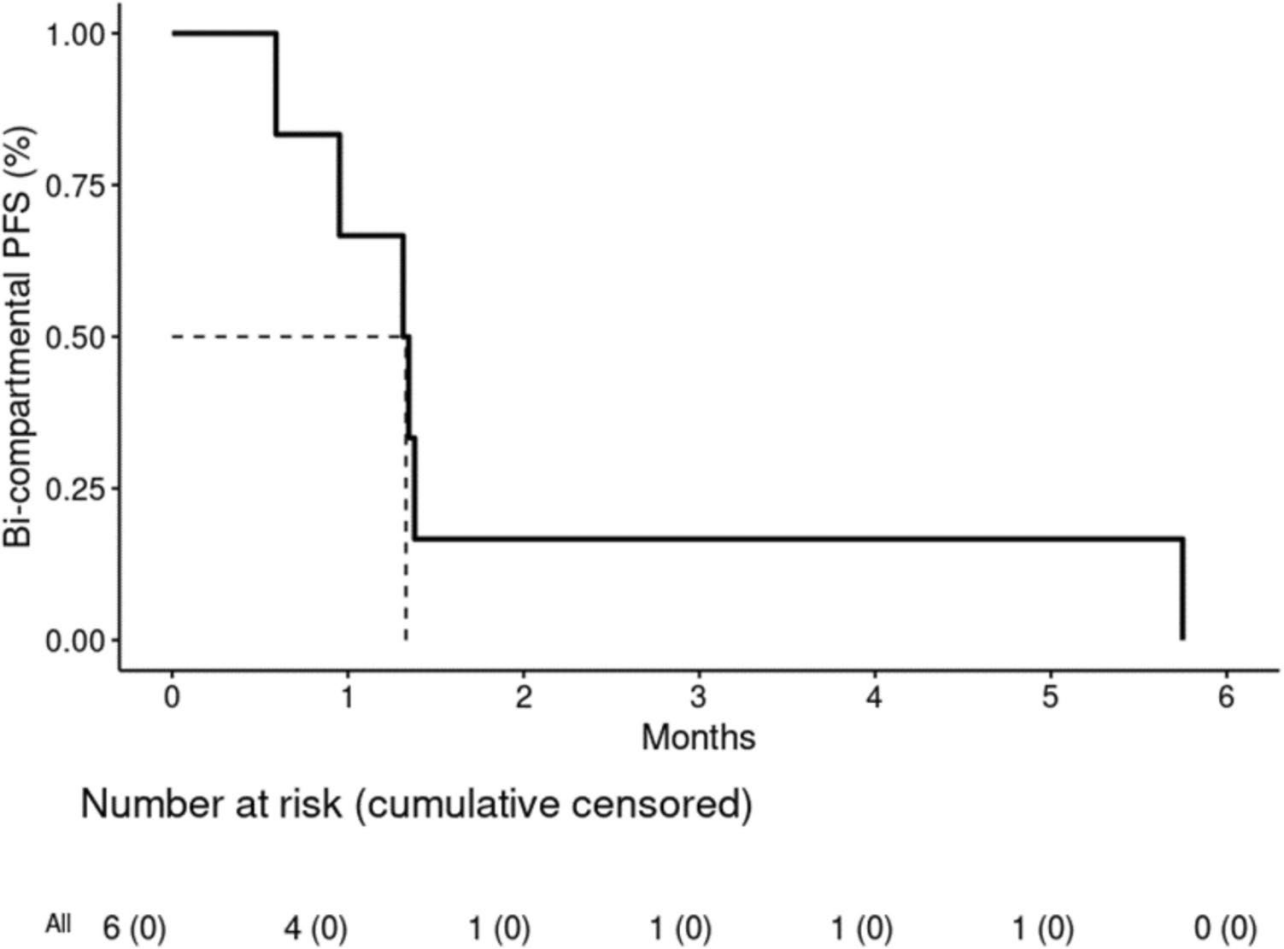
Bi-compartmental progression-free survival

**Fig.2:**
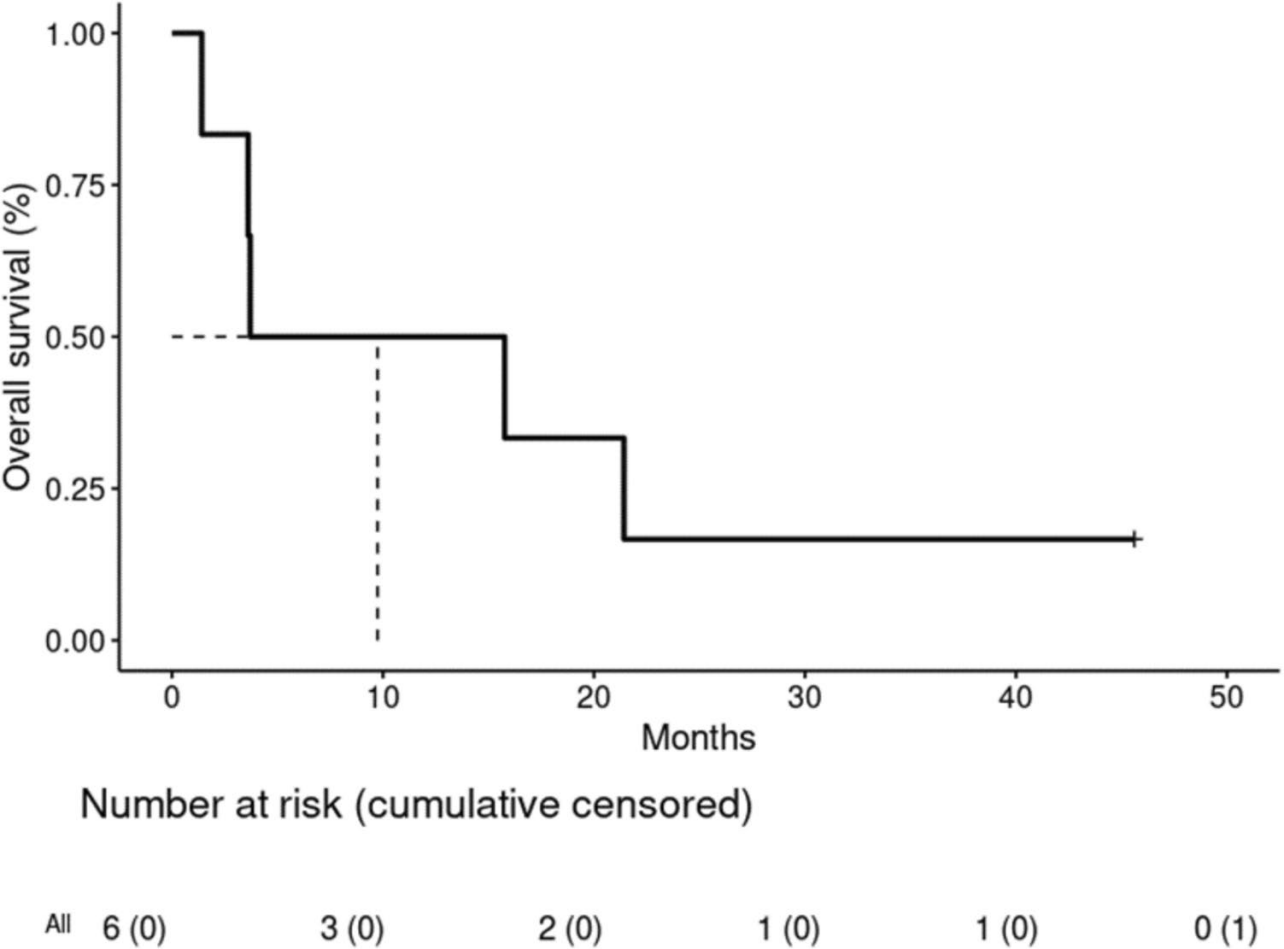
Overall survival

The median bi-compartmental PFS was 1.3 months (95% confidence interval [CI]: 0.95 – NA) (Fig. 1) and the median OS was 9.7 months (95% CI: 3.6 – NA) (Fig. 2). The best observed CNS response by RANO-BM criteria was non-CR/non-progressive disease (PD) in 2 participants (33.3%), of 25- and 6-weeks duration, respectively. One of these patients had an unconfirmed CR (brain magnetic resonance imaging (MRI) confirmed RANO-BM CR 5 months after ending protocol treatment; however, the patient had already started subsequent non-protocol therapy for extracranial progression) (Table 3). The best observed extracranial response by RECIST 1.1 criteria was clinical benefit (stable disease [SD] ≥ 24) weeks in 1 participant (16.7%). No extracranial CR or PR were observed.

**Table 3:** Efficacy

Response by RECIST 1.1 Criteria	N (%)
Objective response rate (95% CI)	0% (0.0% – 45.9%)
Best overall response	
SD SD ≥ 24 weeks	2 (33.3%) 1 (50.0%)
PD	3 (50.0%)
Not Evaluable	1 (16.7%)
CNS Response by RANO-BM Criteria	
Objective response rate (95% CI)	0% (0.0% – 45.9%)
Best overall response	
Non-CR/Non-PD	2 (33.3%)
Non-CR/Non-PD ≥ 24 weeks	1 (50.0%)
Unconfirmed^+^ CR	1 (50.0%)
PD	4 (66.7%)
Bi-compartmental PFS, months (95% CI)	1.3 (0.95—NA)
Overall survival, months (95% CI)	9.7 (3.6—NA)

*CI* confidence interval, *SD* stable disease, *PD* progressive disease, *CNS* central nervous system, *RANO-BM* Response Assessment in Neuro-Oncology Brain Metastases, *CR* complete response, *PFS* progression-free survival

^+^Brain magnetic resonance imaging (MRI) confirmed RANO-BM CR 5 months after ending protocol treatment, however, the patient had already started subsequent non-protocol therapy for extracranial progression

**Fig.3:**
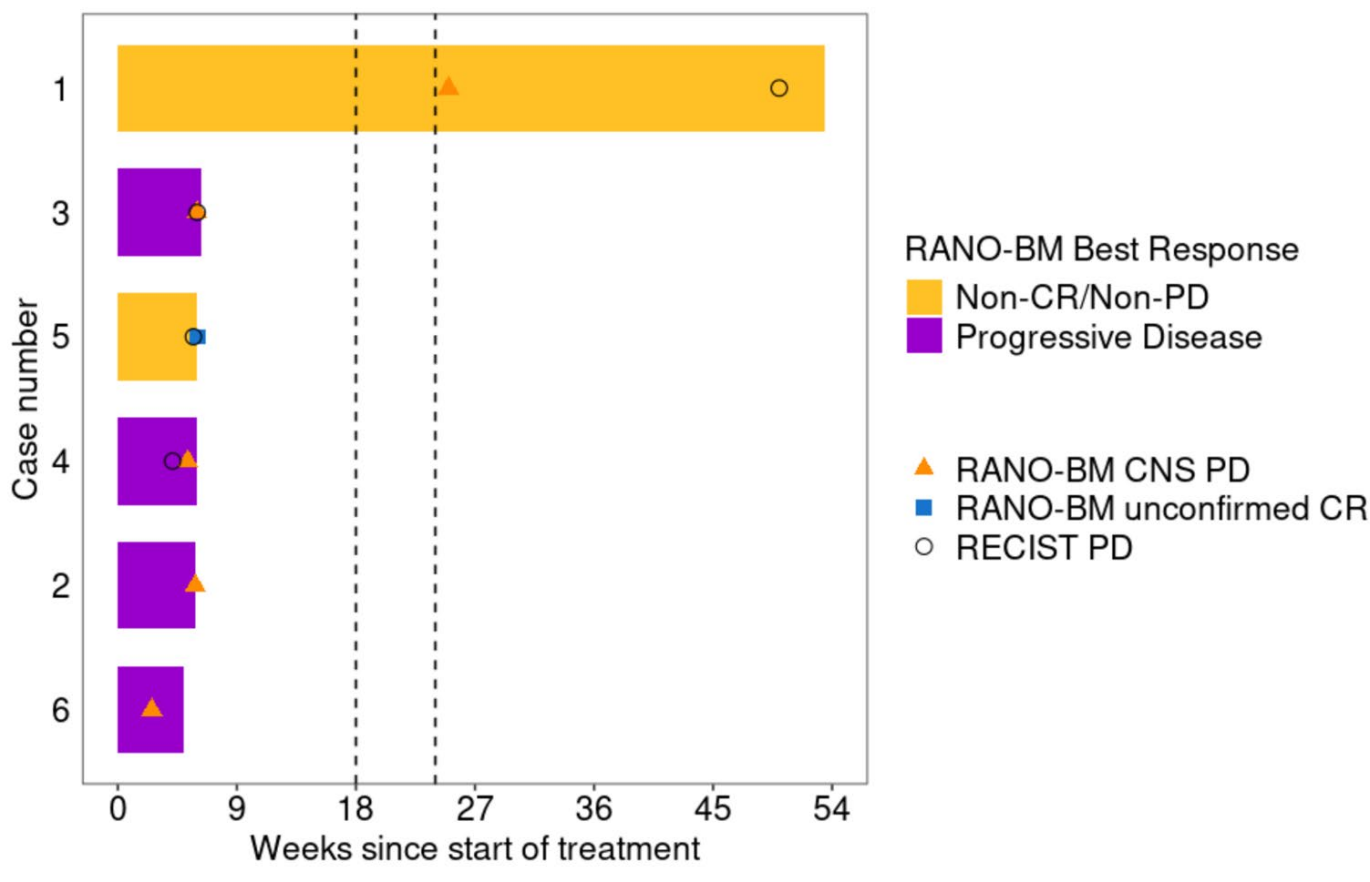
Time on study (swimmer plot). Participant #1 met RANO-BM CNS progression criteria at 25 weeks. Despite this, the decision to continue treatment beyond progression was made due to observed clinical benefit, which is a common practice in certain cases where the overall condition of the patient remains stable or improves. Over time, CNS imaging revealed slight increases in vasogenic edema and peripheral enhancement of two lesions. These findings, coupled with worsening symptoms, prompted surgical intervention. Pathology confirmed that both lesions were consistent with metastatic TNBC, leading to the discontinuation of the study drug. This confirmed that the event at 25 weeks was indeed progressive disease (PD) related to CNS metastases. *RANO-BM* Response Assessment in Neuro-Oncology Brain Metastases, *CR* complete response *PD* progressive disease *CNS* central nervous system

Among 6 patients enrolled, 5 were taken off treatment before or at the first restaging evaluation. Reasons for treatment discontinuation included PD based on RANO-BM and RECIST 1.1 criteria (2 patients; 33.3%), PD based on RANO-BM and clinical evaluation (1 patient, 16.7%), PD based on RANO-BM only (1 patient, 16.7%), and PD based on RECIST 1.1 only (2 patients, 33.3%). Only one patient (case number 1) remained on treatment beyond the first restaging, for a total of 47 weeks (Fig. 3).

For case number 1, investigator-assessed neurological evaluation by NANO score appeared to correspond to prolonged stable disease and worsened at time of disease progression (Supplementary Fig. 1). Likewise, cancer-related symptom severity and interference with daily activities (MDASI-BT) and general health status (EQ-5D) reported by the patient seemed to correlate with treatment duration (Supplementary Figs. 2–4). In the other patients, due to the limited duration of treatment and collected timepoints, no additional PRO analyses could be performed (Supplementary Table 5).

### Exploratory correlative analyses

Next-generation sequencing data were available from 4 participants, in three cases from primary breast tissue and in one case from a nodal metastasis. The PD-L1 score was available for two patients, both of whom were negative. The median tumor mutational burden was 5.3 Mb (range, 2.3–6.1 Mb). A pathogenic *TP53* point mutation, *MCL1* amplification, and *MYC* amplification were each found in 3 samples (75%). All identified mutations and copy number variants (CNVs) are listed in Table 4. Because of the small number of patients and lack of responses, correlations between tumor characteristics and outcomes were not analyzed.

**Table 4:** Mutations identified by next generation sequencing

Sample ID	Tissue	TMB (Mb)	Tier 1–3 mutations	Gene Amplification	PD-L1 status (SP142)
1	primary	5.3	PIK3CA, HRAS	MCL1, MYC	n/a
2	n/a	n/a	n/a	n/a	n/a
3	primary	2.3	TP53, ERBB3	MCL1	n/a
4	n/a	n/a	n/a	n/a	n/a
5	primary	6.1	BRCA2, TP53, RAD50	MYC	< 1%
6	node	5.3	TP53, CHEK2	MCL1, MYC, CCNE1	0

*TMB* tumor mutational burden, *Mb* Mega base, *n/a* not available, *PD-L1* programmed cell death ligand 1

## Discussion

Patients with metastatic TNBC experience worse survival outcomes compared to patients with hormone receptor-positive (HR +) and HER2-positive (HER2 +) breast cancer. This gap is caused both by the aggressive nature of TNBC and the relative lack of effective targeted therapies [36]. In recent years, the approval of ICIs and antibody–drug conjugates (ADCs) for patients with metastatic TNBC has helped to mitigate this disparity. However, outcomes for TNBC patients with brain metastases have remained dismal [13, 37].

We conducted a prospective, single-arm study to evaluate the combination of atezolizumab and SRS in patients with TNBC and brain metastases. When protocol was designed and initiated, ICIs were not included as part of standard-of-care for patients with metastatic TNBC. In the run-in phase of the study, we did not identify major excess toxicities related to concurrent immunotherapy and SRS. There were no grade 3 or higher TRAEs. On the other hand, with the limitation of a small number of patients enrolled, we did not observe compelling activity, with all but one patient discontinuing therapy at the first restaging evaluation due to disease progression. Bi-compartmental PFS by RANO-BM criteria, the primary endpoint, was only 1.3 months, and the median OS was 9.7 months. Our results underscore the poor prognosis and aggressive nature of TNBC that metastasizes to the brain.

Shortly after the initiation of this trial, the treatment landscape for metastatic TNBC began to change rapidly. From March 2019 to September 2021, the combination of atezolizumab with nab-paclitaxel held accelerated U.S. Food and Drug Administration (FDA) approval for the treatment of patients with treatment-naïve locally advanced or metastatic TNBC that was PD-L1-positive according to the companion SP142 assay [38]. Accelerated approval was based on the results of the randomized phase III IMpassion130 trial, which reported that the addition of atezolizumab to nab-paclitaxel resulted in significantly improved PFS in the intention-to-treat (ITT) and PD-L1-positive population [34]. However, in the follow-up IMPassion131 trial, the combination of atezolizumab plus paclitaxel did not result in significant improvement in PFS in the ITT or PD-L1-positive population [39], and the indication for atezolizumab in metastatic TNBC patients was withdrawn.

In 2020, the FDA granted accelerated approval to pembrolizumab in combination with chemotherapy for the treatment of patients with locally recurrent unresectable or metastatic TNBC with a PD-L1 combined positive score (CPS) ≥ 10 with the companion 22C3 diagnostic assay [40]. Approval was based on the results of the randomized phase III KEYNOTE-355 trial, in which patients with treatment-naïve locally recurrent inoperable or metastatic TNBC were randomized 2:1 to receive pembrolizumab or placebo plus chemotherapy (nab-paclitaxel, paclitaxel, or gemcitabine plus carboplatin). The addition of pembrolizumab to chemotherapy produced a significant improvement in median PFS [33] and OS [5], but only among patients with a PD-L1 CPS ≥ 10. Based on these results, pembrolizumab in combination with chemotherapy is currently used as frontline therapy for patients with metastatic TNBC whose tumors express PD-L1.

The present study of atezolizumab in combination with SRS for metastatic TNBC patients with brain metastases was closed early after atezolizumab and pembrolizumab received FDA approval for metastatic TNBC, as the approvals resulted in changes in standard of care and significantly slowed study accrual.

The efficacy of ICIs has not been thoroughly explored in TNBC patients with brain metastases. Patients with active CNS metastases were excluded from KEYNOTE-355, though patients with previously treated, stable brain metastases were eligible. However, the trial only included 26 participants with stable brain metastases, and the efficacy of each treatment arm in patients with brain metastases was not evaluated [33]. The IMPassion130 trial included 61 participants with brain metastases. Among these participants, the median PFS was 4.9 months on atezolizumab plus chemotherapy versus 4.4 months on placebo plus chemotherapy (hazard ratio [HR] = 0.86; 95% CI: 0.50–1.49) [34], and the median OS was 14.3 months on atezolizumab plus chemotherapy versus 16.2 months on placebo plus chemotherapy (HR = 1.16; 95% CI: 0.66–2.04) [41].

In our cohort of patients, no patient had elevated tumor mutational burden (all samples < 10 Mb). In agreement with other meta-analyses [42, 43], the two most frequently mutated genes, *TP53* and *PIK3CA*, were also identified in three and one of four patients, respectively. Regarding CNVs, three of four patients presented with *MYC* and *MCL1* amplifications, respectively. *MYC*, which is involved in cell cycle progression, apoptosis, and cellular transformation, and *MCL1*, which is involved in apoptosis and cell survival, are often amplified in brain metastatic samples [44]. Because of the sample size, correlations with clinical outcomes were not possible.

Our study’s major limitation is the small sample size, with only six patients enrolled. Brain metastasis from TNBC has been largely unexplored as patients have been actively excluded from most clinical trials. It is imperative, based on the high rate and mortality of CNS disease in TNBC, to continue exploring multi-disciplinary approaches with various therapeutic combinations to achieve better results. In our study, subjects received single-agent ICI. Several studies with immunotherapy in TNBC have subsequently demonstrated superior overall response rates of checkpoint inhibitors in combination with chemotherapy, rather than as single agent [33, 34, 45]. We may speculate that a combination strategy of chemotherapy plus ICI could have led to better results in combination with SRS. Finally, accumulating data suggest potential intracranial efficacy of antibody–drug conjugates (ADCs) such as sacituzumab govitecan and trastuzumab deruxtecan [46–51]. For instance, a phase 0 window of opportunity trial that included 13 patients with breast cancer brain metastases showed that sacituzumab govitecan achieves therapeutically relevant concentrations of its payload (SN-38) at 150-fold mean IC50s. Among these patients, the observed PFS was 8 months (range: 2–26.5) and the observed OS was 35.2 months (range: 2.7–37) [49]. Ongoing clinical trials are testing combinations of ADCs with ICIs, which could be especially appealing for breast cancer patients with CNS disease. A single-arm phase II trial evaluated adebrelimab (an anti-PD-L1 ICI) in combination with bevacizumab and cisplatin/carboplatin in TNBC patients with active brain metastases. Among 35 enrolled participants, the CNS-ORR was 77.1% (confirmed CNS-ORR 71.4%) and the CNS-PFS was 10 months (95% CI: 7.4–12.6). In addition, the median PFS was 7.6 months (95% CI: 5.7–11.5) and the median OS was 16 months (95% CI: 11.7 – Not Reached [NR]) [52].

The development of bispecific antibodies also shows some potential for the treatment of TNBC patients with brain metastases. Pumitamig is a bispecific antibody that targets VEGF and PD-L1. In a phase Ib/II trial, 42 patients with locally advanced or metastatic TNBC were treated with first-line pumitamig in combination with nab-paclitaxel. After a median follow-up of 18.1 months, the confirmed ORR was 73.8%, the disease control rate was 95.2%, and the median PFS was 13.5 months (95% CI: 9.4–18.1). Phase II (NCT06449222) and phase III (NCT06419621) trials are underway, and it will be interesting to see if there is activity in patients with CNS metastases [53]. In addition, ivonescimab is a tetrameric bispecific antibody that targets VEGF and PD-1. In an open-label phase II trial, 30 patients with locally advanced unresectable or metastatic TNBC received ivonescimab in combination with paclitaxel or nab-paclitaxel. After a median follow-up of 7.2 months, the investigator-assessed ORR was 72.4%, the disease control rate was 100%, and the 6-month PFS was 68.4% (95% CI: 44.3%—83.8%0. The median PFS and OS were not yet mature [54].

In summary, in this small prospective clinical trial, we did not identify new safety signals or preliminary evidence of synergy between concurrent SRS and atezolizumab in TNBC patients with brain metastases. Our results do not rule out a potential contribution of ICI therapy in patients with brain metastases, but suggest that combination strategies will be needed to maximize their promise. Finally, all patients in our study received SRS, yet still experienced poor outcomes, with rapid disease progression and short OS. This highlights the tremendous unmet medical need to develop effective multidisciplinary approaches for the management of patients with brain metastases from TNBC.

## Supplementary Information

Below is the link to the electronic supplementary material.

Supplementary file1 (PDF 246 KB)

## Data Availability

Deidentified individual-level patient data that support the findings of this study are available from the corresponding author on reasonable request.

## References

[1] Foulkes WD, Smith IE, Reis-Filho JS (2010) Triple-negative breast cancer. N Engl J Med 363(20):1938–1948. 10.1056/NEJMra1001389 21067385 10.1056/NEJMra1001389

[2] Lin NU, Vanderplas A, Hughes ME, Theriault RL, Edge SB, Wong YN, Blayney DW, Niland JC, Winer EP, Weeks JC (2012) Clinicopathologic features, patterns of recurrence, and survival among women with triple-negative breast cancer in the National Comprehensive Cancer Network. Cancer 118(22):5463–5472. 10.1002/cncr.27581 22544643 10.1002/cncr.27581 PMC3611659

[3] Dent R, Trudeau M, Pritchard KI, Hanna WM, Kahn HK, Sawka CA, Lickley LA, Rawlinson E, Sun P, Narod SA (2007) Triple-negative breast cancer: clinical features and patterns of recurrence. Clin Cancer Res 13(15 Pt 1):4429–4434. 10.1158/1078-0432.CCR-06-3045 17671126 10.1158/1078-0432.CCR-06-3045

[4] Skinner KE, Haiderali A, Huang M, Schwartzberg LS (2021) Real-world effectiveness outcomes in patients diagnosed with metastatic triple-negative breast cancer. Future Oncol 17(8):931–941. 10.2217/fon-2020-1021 33207944 10.2217/fon-2020-1021

[5] Cortes J, Rugo HS, Cescon DW, Im SA, Yusof MM, Gallardo C, Lipatov O, Barrios CH, Perez-Garcia J, Iwata H, Masuda N, Torregroza Otero M, Gokmen E, Loi S, Guo Z, Zhou X, Karantza V, Pan W, Schmid P (2022) Pembrolizumab plus chemotherapy in advanced triple-negative breast cancer. N Engl J Med 387(3):217–226. 10.1056/NEJMoa2202809. (**Investigators K-**) 35857659 10.1056/NEJMoa2202809

[6] Lin NU, Claus E, Sohl J, Razzak AR, Arnaout A, Winer EP (2008) Sites of distant recurrence and clinical outcomes in patients with metastatic triple-negative breast cancer: high incidence of central nervous system metastases. Cancer 113(10):2638–2645. 10.1002/cncr.23930 18833576 10.1002/cncr.23930 PMC2835546

[7] Kennecke H, Yerushalmi R, Woods R, Cheang MC, Voduc D, Speers CH, Nielsen TO, Gelmon K (2010) Metastatic behavior of breast cancer subtypes. J Clin Oncol 28(20):3271–3277. 10.1200/JCO.2009.25.9820 20498394 10.1200/JCO.2009.25.9820

[8] Sammons SL, Sanglier T, Leone JP, Erick TK, Lambert P, Montemurro F, Poppe R, Restuccia E, Tolaney SM, Lin NU (2025) Prevalence by therapy line and incidence of breast cancer brain metastases in 18,075 patients. J Natl Cancer Inst. 10.1093/jnci/djaf048 40163696 10.1093/jnci/djaf048

[9] Lin NU (2014) Targeted therapies in brain metastases. Curr Treat Options Neurol 16(1):276. 10.1007/s11940-013-0276-z 24353011 10.1007/s11940-013-0276-z PMC3895218

[10] Riecke K, Muller V, Neunhoffer T, Park-Simon TW, Weide R, Polasik A, Schmidt M, Puppe J, Mundhenke C, Lubbe K, Hesse T, Thill M, Wuerstlein R, Denkert C, Decker T, Fehm T, Nekljudova V, Rey J, Loibl S, Laakmann E, Witzel I (2023) Long-term survival of breast cancer patients with brain metastases: subanalysis of the BMBC registry. ESMO Open 8(3):101213. 10.1016/j.esmoop.2023.101213 37075697 10.1016/j.esmoop.2023.101213 PMC10265610

[11] Dawood S, Broglio K, Esteva FJ, Yang W, Kau SW, Islam R, Albarracin C, Yu TK, Green M, Hortobagyi GN, Gonzalez-Angulo AM (2009) Survival among women with triple receptor-negative breast cancer and brain metastases. Ann Oncol 20(4):621–627. 10.1093/annonc/mdn682 19150943 10.1093/annonc/mdn682 PMC2722369

[12] Sperduto PW, Kased N, Roberge D, Chao ST, Shanley R, Luo X, Sneed PK, Suh J, Weil RJ, Jensen AW, Brown PD, Shih HA, Kirkpatrick J, Gaspar LE, Fiveash JB, Chiang V, Knisely JP, Sperduto CM, Lin N, Mehta M (2013) The effect of tumor subtype on the time from primary diagnosis to development of brain metastases and survival in patients with breast cancer. J Neurooncol 112(3):467–472. 10.1007/s11060-013-1083-9 23462853 10.1007/s11060-013-1083-9

[13] Sperduto PW, Mesko S, Li J, Cagney D, Aizer A, Lin NU, Nesbit E, Kruser TJ, Chan J, Braunstein S, Lee J, Kirkpatrick JP, Breen W, Brown PD, Shi D, Shih HA, Soliman H, Sahgal A, Shanley R, Sperduto W, Lou E, Everett A, Boggs DH, Masucci L, Roberge D, Remick J, Plichta K, Buatti JM, Jain S, Gaspar LE, Wu CC, Wang TJC, Bryant J, Chuong M, Yu J, Chiang V, Nakano T, Aoyama H, Mehta MP (2020) Beyond an updated graded prognostic assessment (Breast GPA): a prognostic index and trends in treatment and survival in breast cancer brain metastases from 1985 to today. Int J Radiat Oncol Biol Phys 107(2):334–343. 10.1016/j.ijrobp.2020.01.051 32084525 10.1016/j.ijrobp.2020.01.051 PMC7276246

[14] Sharabi AB, Nirschl CJ, Kochel CM, Nirschl TR, Francica BJ, Velarde E, Deweese TL, Drake CG (2015) Stereotactic radiation therapy augments antigen-specific PD-1-mediated antitumor immune responses via cross-presentation of tumor antigen. Cancer Immunol Res 3(4):345–355. 10.1158/2326-6066.CIR-14-0196 25527358 10.1158/2326-6066.CIR-14-0196 PMC4390444

[15] Deng L, Liang H, Burnette B, Beckett M, Darga T, Weichselbaum RR, Fu YX (2014) Irradiation and anti-PD-L1 treatment synergistically promote antitumor immunity in mice. J Clin Invest 124(2):687–695. 10.1172/JCI67313 24382348 10.1172/JCI67313 PMC3904601

[16] Kiess AP, Wolchok JD, Barker CA, Postow MA, Tabar V, Huse JT, Chan TA, Yamada Y, Beal K (2015) Stereotactic radiosurgery for melanoma brain metastases in patients receiving ipilimumab: safety profile and efficacy of combined treatment. Int J Radiat Oncol Biol Phys 92(2):368–375. 10.1016/j.ijrobp.2015.01.004 25754629 10.1016/j.ijrobp.2015.01.004 PMC4955924

[17] Schoenfeld JD, Mahadevan A, Floyd SR, Dyer MA, Catalano PJ, Alexander BM, McDermott DF, Kaplan ID (2015) Ipilmumab and cranial radiation in metastatic melanoma patients: a case series and review. J Immunother Cancer 3(1):50. 10.1186/s40425-015-0095-8 26672895 10.1186/s40425-015-0095-8 PMC4678639

[18] Ahmed KA, Stallworth DG, Kim Y, Johnstone PA, Harrison LB, Caudell JJ, Yu HH, Etame AB, Weber JS, Gibney GT (2016) Clinical outcomes of melanoma brain metastases treated with stereotactic radiation and anti-PD-1 therapy. Ann Oncol 27(3):434–441. 10.1093/annonc/mdv622 26712903 10.1093/annonc/mdv622

[19] Theelen W, Peulen HMU, Lalezari F, van der Noort V, de Vries JF, Aerts J, Dumoulin DW, Bahce I, Niemeijer AN, de Langen AJ, Monkhorst K, Baas P (2019) Effect of pembrolizumab after stereotactic body radiotherapy vs pembrolizumab alone on tumor response in patients with advanced non-small cell lung cancer: results of the PEMBRO-RT phase 2 randomized clinical trial. JAMA Oncol 5(9):1276–1282. 10.1001/jamaoncol.2019.1478 31294749 10.1001/jamaoncol.2019.1478 PMC6624814

[20] Ahmed KA, Kim S, Arrington J, Naghavi AO, Dilling TJ, Creelan BC, Antonia SJ, Caudell JJ, Harrison LB, Sahebjam S, Gray JE, Etame AB, Johnstone PA, Yu M, Perez BA (2017) Outcomes targeting the PD-1/PD-L1 axis in conjunction with stereotactic radiation for patients with non-small cell lung cancer brain metastases. J Neurooncol 133(2):331–338. 10.1007/s11060-017-2437-5 28466250 10.1007/s11060-017-2437-5

[21] Chen L, Douglass J, Kleinberg L, Ye X, Marciscano AE, Forde PM, Brahmer J, Lipson E, Sharfman W, Hammers H, Naidoo J, Bettegowda C, Lim M, Redmond KJ (2018) Concurrent immune checkpoint inhibitors and stereotactic radiosurgery for brain metastases in non-small cell lung cancer, melanoma, and renal cell carcinoma. Int J Radiat Oncol Biol Phys 100(4):916–925. 10.1016/j.ijrobp.2017.11.041 29485071 10.1016/j.ijrobp.2017.11.041

[22] Dagoglu N, Karaman S, Caglar HB, Oral EN (2019) Abscopal effect of radiotherapy in the immunotherapy era: systematic review of reported cases. Cureus 11(2):e4103. 10.7759/cureus.4103 31057997 10.7759/cureus.4103 PMC6476623

[23] Wolff AC, Somerfield MR, Dowsett M, Hammond MEH, Hayes DF, McShane LM, Saphner TJ, Spears PA, Allison KH (2023) Human epidermal growth factor receptor 2 testing in breast cancer: ASCO-College of American Pathologists guideline update. J Clin Oncol 41(22):3867–3872. 10.1200/JCO.22.02864 37284804 10.1200/JCO.22.02864

[24] Eisenhauer EA, Therasse P, Bogaerts J, Schwartz LH, Sargent D, Ford R, Dancey J, Arbuck S, Gwyther S, Mooney M, Rubinstein L, Shankar L, Dodd L, Kaplan R, Lacombe D, Verweij J (2009) New response evaluation criteria in solid tumours: revised RECIST guideline (version 1.1). Eur J Cancer 45(2):228–247. 10.1016/j.ejca.2008.10.026 19097774 10.1016/j.ejca.2008.10.026

[25] Okada H, Weller M, Huang R, Finocchiaro G, Gilbert MR, Wick W, Ellingson BM, Hashimoto N, Pollack IF, Brandes AA, Franceschi E, Herold-Mende C, Nayak L, Panigrahy A, Pope WB, Prins R, Sampson JH, Wen PY, Reardon DA (2015) Immunotherapy response assessment in neuro-oncology: a report of the RANO working group. Lancet Oncol 16(15):e534–e542. 10.1016/S1470-2045(15)00088-1 26545842 10.1016/S1470-2045(15)00088-1 PMC4638131

[26] Wolchok JD, Hoos A, O’Day S, Weber JS, Hamid O, Lebbe C, Maio M, Binder M, Bohnsack O, Nichol G, Humphrey R, Hodi FS (2009) Guidelines for the evaluation of immune therapy activity in solid tumors: immune-related response criteria. Clin Cancer Res 15(23):7412–7420. 10.1158/1078-0432.CCR-09-1624 19934295 10.1158/1078-0432.CCR-09-1624

[27] Nayak L, DeAngelis LM, Brandes AA, Peereboom DM, Galanis E, Lin NU, Soffietti R, Macdonald DR, Chamberlain M, Perry J, Jaeckle K, Mehta M, Stupp R, Muzikansky A, Pentsova E, Cloughesy T, Iwamoto FM, Tonn JC, Vogelbaum MA, Wen PY, van den Bent MJ, Reardon DA (2017) The neurologic assessment in neuro-oncology (NANO) scale: a tool to assess neurologic function for integration into the Response Assessment in Neuro-Oncology (RANO) criteria. Neuro Oncol 19(5):625–635. 10.1093/neuonc/nox029 28453751 10.1093/neuonc/nox029 PMC5464449

[28] Armstrong TS, Mendoza T, Gning I, Coco C, Cohen MZ, Eriksen L, Hsu MA, Gilbert MR, Cleeland C (2006) Validation of the M.D. anderson symptom inventory brain tumor module (MDASI-BT). J Neurooncol 80(1):27–35. 10.1007/s11060-006-9135-z 16598415 10.1007/s11060-006-9135-z

[29] Herdman M, Gudex C, Lloyd A, Janssen M, Kind P, Parkin D, Bonsel G, Badia X (2011) Development and preliminary testing of the new five-level version of EQ-5D (EQ-5D-5L). Qual Life Res 20(10):1727–1736. 10.1007/s11136-011-9903-x 21479777 10.1007/s11136-011-9903-x PMC3220807

[30] Lin NU, Lee EQ, Aoyama H, Barani IJ, Barboriak DP, Baumert BG, Bendszus M, Brown PD, Camidge DR, Chang SM, Dancey J, de Vries EG, Gaspar LE, Harris GJ, Hodi FS, Kalkanis SN, Linskey ME, Macdonald DR, Margolin K, Mehta MP, Schiff D, Soffietti R, Suh JH, van den Bent MJ, Vogelbaum MA, Wen PY (2015) Response assessment criteria for brain metastases: proposal from the RANO group. Lancet Oncol 16(6):e270-278. 10.1016/S1470-2045(15)70057-4 26065612 10.1016/S1470-2045(15)70057-4

[31] Anders C, Deal AM, Abramson V, Liu MC, Storniolo AM, Carpenter JT, Puhalla S, Nanda R, Melhem-Bertrandt A, Lin NU, Kelly Marcom P, Van Poznak C, Stearns V, Melisko M, Smith JK, Karginova O, Parker J, Berg J, Winer EP, Peterman A, Prat A, Perou CM, Wolff AC, Carey LA (2014) TBCRC 018: phase II study of iniparib in combination with irinotecan to treat progressive triple negative breast cancer brain metastases. Breast Cancer Res Treat 146(3):557–566. 10.1007/s10549-014-3039-y 25001612 10.1007/s10549-014-3039-y PMC4112043

[32] Dyer MA, Kelly PJ, Chen YH, Pinnell NE, Claus EB, Lee EQ, Weiss SE, Arvold ND, Lin NU, Alexander BM (2012) Importance of extracranial disease status and tumor subtype for patients undergoing radiosurgery for breast cancer brain metastases. Int J Radiat Oncol Biol Phys 83(4):e479-486. 10.1016/j.ijrobp.2012.01.054 22704705 10.1016/j.ijrobp.2012.01.054

[33] Cortes J, Cescon DW, Rugo HS, Nowecki Z, Im SA, Yusof MM, Gallardo C, Lipatov O, Barrios CH, Holgado E, Iwata H, Masuda N, Otero MT, Gokmen E, Loi S, Guo Z, Zhao J, Aktan G, Karantza V, Schmid P, Investigators K- (2020) Pembrolizumab plus chemotherapy versus placebo plus chemotherapy for previously untreated locally recurrent inoperable or metastatic triple-negative breast cancer (KEYNOTE-355): a randomised, placebo-controlled, double-blind, phase 3 clinical trial. Lancet 396(10265):1817–1828. 10.1016/S0140-6736(20)32531-9 33278935 10.1016/S0140-6736(20)32531-9

[34] Schmid P, Adams S, Rugo HS, Schneeweiss A, Barrios CH, Iwata H, Dieras V, Hegg R, Im SA, Shaw Wright G, Henschel V, Molinero L, Chui SY, Funke R, Husain A, Winer EP, Loi S, Emens LA, Investigators IMT (2018) Atezolizumab and Nab-Paclitaxel in advanced triple-negative breast cancer. N Engl J Med 379(22):2108–2121. 10.1056/NEJMoa1809615 30345906 10.1056/NEJMoa1809615

[35] Almeida ND, Kuo C, Schrand TV, Rupp J, Madhugiri VS, Goulenko V, Shekher R, Shah C, Prasad D (2024) Stereotactic radiosurgery for intracranial breast metastases: a systematic review and meta-analysis. Cancers (Basel). 10.3390/cancers16203551 39456645 10.3390/cancers16203551 PMC11506708

[36] Waks AG, Winer EP (2019) Breast cancer treatment: a review. JAMA 321(3):288–300. 10.1001/jama.2018.19323 30667505 10.1001/jama.2018.19323

[37] Pasquier D, Darlix A, Louvel G, Fraisse J, Jacot W, Brain E, Petit A, Mouret-Reynier MA, Goncalves A, Dalenc F, Deluche E, Fresnel JS, Augereau P, Ferrero JM, Geffrelot J, Fumet JD, Lecouillard I, Cottu P, Petit T, Uwer L, Jouannaud C, Leheurteur M, Dieras V, Robain M, Mouttet-Audouard R, Bachelot T, Courtinard C (2020) Treatment and outcomes in patients with central nervous system metastases from breast cancer in the real-life ESME MBC cohort. Eur J Cancer 125:22–30. 10.1016/j.ejca.2019.11.001 31835235 10.1016/j.ejca.2019.11.001

[38] American Society of Clinical Oncology. Update on U.S. Indication for Atezolizumab in PD-L1–Positive Metastatic Triple-Negative Breast Cancer (2021).

[39] Miles D, Gligorov J, Andre F, Cameron D, Schneeweiss A, Barrios C, Xu B, Wardley A, Kaen D, Andrade L, Semiglazov V, Reinisch M, Patel S, Patre M, Morales L, Patel SL, Kaul M, Barata T, O’Shaughnessy J (2021) Primary results from IMpassion131, a double-blind, placebo-controlled, randomised phase III trial of first-line paclitaxel with or without atezolizumab for unresectable locally advanced/metastatic triple-negative breast cancer. Ann Oncol 32(8):994–1004. 10.1016/j.annonc.2021.05.801 34219000 10.1016/j.annonc.2021.05.801

[40] U.S. Food and Drug Administration. FDA grants accelerated approval to pembrolizumab for locally recurrent unresectable or metastatic triple negative breast cancer (2020).

[41] Emens LA, Adams S, Barrios CH, Dieras V, Iwata H, Loi S, Rugo HS, Schneeweiss A, Winer EP, Patel S, Henschel V, Swat A, Kaul M, Molinero L, Patel S, Chui SY, Schmid P (2021) First-line atezolizumab plus nab-paclitaxel for unresectable, locally advanced, or metastatic triple-negative breast cancer: IMpassion130 final overall survival analysis. Ann Oncol 32(8):983–993. 10.1016/j.annonc.2021.05.355 34272041 10.1016/j.annonc.2021.05.355

[42] Nguyen TT, Hamdan D, Angeli E, Feugeas JP, Le QV, Pamoukdjian F, Bousquet G (2023) Genomics of breast cancer brain metastases: a meta-analysis and therapeutic implications. Cancers (Basel). 10.3390/cancers15061728 36980614 10.3390/cancers15061728 PMC10046845

[43] Morgan AJ, Giannoudis A, Palmieri C (2021) The genomic landscape of breast cancer brain metastases: a systematic review. Lancet Oncol 22(1):e7–e17. 10.1016/S1470-2045(20)30556-8 33387511 10.1016/S1470-2045(20)30556-8

[44] Campbell BK, Gao Z, Corcoran NM, Stylli SS, Hovens CM (2022) Molecular mechanisms driving the formation of brain metastases. Cancers (Basel). 10.3390/cancers14194963 36230886 10.3390/cancers14194963 PMC9563727

[45] Winer EP, Lipatov O, Im SA, Goncalves A, Munoz-Couselo E, Lee KS, Schmid P, Tamura K, Testa L, Witzel I, Ohtani S, Turner N, Zambelli S, Harbeck N, Andre F, Dent R, Zhou X, Karantza V, Mejia J, Cortes J (2021) Pembrolizumab versus investigator-choice chemotherapy for metastatic triple-negative breast cancer (KEYNOTE-119): a randomised, open-label, phase 3 trial. Lancet Oncol 22(4):499–511. 10.1016/S1470-2045(20)30754-3 33676601 10.1016/S1470-2045(20)30754-3

[46] Kabraji S, Ni J, Sammons S, Li T, Van Swearingen AED, Wang Y, Pereslete A, Hsu L, DiPiro PJ, Lascola C, Moore H, Hughes M, Raghavendra AS, Gule-Monroe M, Murthy RK, Winer EP, Anders CK, Zhao JJ, Lin NU (2023) Preclinical and clinical efficacy of Trastuzumab Deruxtecan in breast cancer brain metastases. Clin Cancer Res 29(1):174–182. 10.1158/1078-0432.CCR-22-1138 36074155 10.1158/1078-0432.CCR-22-1138 PMC9811155

[47] Perez-Garcia JM, Vaz Batista M, Cortez P, Ruiz-Borrego M, Cejalvo JM, de la Haba-Rodriguez J, Garrigos L, Racca F, Servitja S, Blanch S, Gion M, Nave M, Fernandez-Abad M, Martinez-Bueno A, Llombart-Cussac A, Sampayo-Cordero M, Malfettone A, Cortes J, Braga S (2023) Trastuzumab deruxtecan in patients with central nervous system involvement from HER2-positive breast cancer: the DEBBRAH trial. Neuro Oncol 25(1):157–166. 10.1093/neuonc/noac144 35639825 10.1093/neuonc/noac144 PMC9825345

[48] Bartsch R, Berghoff AS, Furtner J, Marhold M, Bergen ES, Roider-Schur S, Starzer AM, Forstner H, Rottenmanner B, Dieckmann K, Bago-Horvath Z, Haslacher H, Widhalm G, Ilhan-Mutlu A, Minichsdorfer C, Fuereder T, Szekeres T, Oehler L, Gruenberger B, Singer CF, Weltermann A, Puhr R, Preusser M (2022) Trastuzumab deruxtecan in HER2-positive breast cancer with brain metastases: a single-arm, phase 2 trial. Nat Med 28(9):1840–1847. 10.1038/s41591-022-01935-8 35941372 10.1038/s41591-022-01935-8 PMC9499862

[49] Balinda HU, Kelly WJ, Kaklamani VG, Lathrop KI, Canola MM, Ghamasaee P, Sareddy GR, Michalek J, Gilbert AR, Surapaneni P, Tiziani S, Pandey R, Chiou J, Lodi A, Floyd JR 2nd, Brenner AJ (2024) Sacituzumab Govitecan in patients with breast cancer brain metastases and recurrent glioblastoma: a phase 0 window-of-opportunity trial. Nat Commun 15(1):6707. 10.1038/s41467-024-50558-9 39112464 10.1038/s41467-024-50558-9 PMC11306739

[50] Dannehl D, Jakob D, Mergel F, Estler A, Engler T, Volmer L, Frevert ML, Matovina S, Englisch A, Tegeler CM, Rohner A, Seller A, Hahn M, Pfister K, Fink A, Popp I, Lorenz S, Tabatabai G, Juhasz-Boss I, Janni W, Brucker S, Taran FA, Hartkopf A, Schaffler H (2024) The efficacy of sacituzumab govitecan and trastuzumab deruxtecan on stable and active brain metastases in metastatic breast cancer patients-a multicenter real-world analysis. ESMO Open 9(5):102995. 10.1016/j.esmoop.2024.102995 38636292 10.1016/j.esmoop.2024.102995 PMC11039313

[51] Grinda T, Morganti S, Hsu L, Yoo TK, Kusmick RJ, Aizer AA, Giordano A, Leone JP, Hughes M, Tolaney SM, Lin NU, Sammons SL (2025) Real-world outcomes with sacituzumab govitecan among breast cancer patients with central nervous system metastases. NPJ Breast Cancer 11(1):22. 10.1038/s41523-025-00736-9 40038301 10.1038/s41523-025-00736-9 PMC11880407

[52] Li T, Wang B, Tao Z, Zhao M, Wang L, Jin J, Zhao Y, Gong C, Cao J, Miao H, Wang J, Hu X, Zhang J (2025) A phase II clinical study of adebrelimab and bevacizumab combined with cisplatin/carboplatin in triple-negative breast cancer patients with brain metastases. J Clin Oncol 43(16_suppl):1018–1018. 10.1200/JCO.2025.43.16_suppl.1018 10.1200/JCO-25-02021 PMC13557479 41779972

[53] Wu J, Zhang J, Tong Z, Zhang Q, Wang Y, Cheng Q, Chen X, Li Z, Yin Y, Du Y, Meng Y (2025) Abstract PS3-08: interim overall survival of patients with locally advanced or metastatic triple-negative breast cancer treated with first line PM8002/BNT327 in combination with nab-paclitaxel in phase Ib/II study. Clin Cancer Res 31(12_Supplement):PS3-08-PS03-08. 10.1158/1557-3265.SABCS24-PS3-08

[54] Ouyang Q, Wang X, Tian C, Shao X, Huang J, Chen ZH, wang Y, Sun T, Yi T, Yu X, Wang ZM, Li B, Xia Y (2024) 347MO the safety and efficacy of ivonescimab in combination with chemotherapy as first-line (1L) treatment for triple-negative breast cancer (TNBC). Ann Oncol 35:S360–S361. 10.1016/j.annonc.2024.08.295

