# Supplementary material for: A phase II study of atezolizumab in combination with stereotactic radiation for patients with triple-negative breast cancer and brain metastasis
Source: Breast Cancer Res Treat. 2026 Mar 7;216(2):24. doi: 10.1007/s10549-026-07932-6 (PMC12967526; doi:10.1007/s10549-026-07932-6)
Supplement: Supplementary file 1 — Supplementary file1 (PDF 246 KB) [file 10549_2026_7932_MOESM1_ESM.pdf]

Supplement

Article: A phase II study of atezolizumab in combination with stereotactic radiation for patients with triple-negative breast cancer and brain metastasis

Journal: Breast Cancer Research and Treatment

Authors: Antonio Giordano, MD, PhD[1,2]; Noah Graham, MB[1]; Ayal A. Aizer, MD[1,2,3]; Nabihah Tayob, PhD[1,2]; Alyssa M. Pereslete, MD[1]; Jonathan D. Schoenfeld, MD, MPH[1,2]; Jose Pablo Leone, MD[1,2], Raechel Davis, MS[1], Timothy K. Erick, PhD[1]; Erica L. Mayer, MD, MPH[1,2];Eric P. Winer, MD[4,5], Ian Krop, MD, PhD[4,5]; Sara M. Tolaney, MD, MPH[1,2]; Nancy U. Lin, MD[1,2]

Corresponding author:

Nancy U. Lin, MD

Dana-Farber Cancer Institute

450 Brookline Avenue

Boston, MA 02215

Nancy_Lin@dfci.harvard.edu

Supplementary Table 1. Patient disposition at time of data cutoff.

**Characteristic**	**N (%)**
Dates of Registration (range)	5/11/2018 – 10/24/2019
Date of Data Cutoff	1/7/2025
Patient disposition at time of data cutoff	
Off protocol therapy	6 (100.0%)
Study status at time of data cutoff	
Dead	5 (83.3%)
Lost to follow-up	1 (16.7%)
Reason treatment ended	
CNS PD per RANO-BM and extracranial PD per RECIST 1.1	2 (33.3%)
CNS PD per RANO-BM and clinical evaluation	1 (16.7%)
CNS PD per RANO-BM, only	1 (16.7%)
Extracranial PD per RECIST 1.1	2 (33.3%)

Abbreviations: CNS, central nervous system; PD, progressive disease; RANO-BM, Response Assessment in Neuro-Oncology Brain Metastases.

Supplementary Table 2. Atezolizumab treatment details

**Characteristic**	**N (%)**
Number of cycles per patient, median (range)	2 (2 – 16)
Total number of cycles administered	
2	5 (83.3%)
16	1 (16.7%)
Number of patients with dose hold	0 (0.0%)
Number of patients with dose reduction	0 (0.0%)

Supplementary Table 3. Stereotactic radiosurgery details

Characteristic	N (%)
Number of metastases irradiated	
1	1 (16.7%)
2	2 (33.3%)
4	1 (16.7%)
5	2 (33.3%)
Dose administered, Gy [a]	
20	6 (100.0%)
Fractions administered [a]	
1	6 (100.0%)
Volume of PTV receiving the prescription	
dose, % [a]	3 (50.0%)
99.0	2 (33.3%)
99.1	1 (16.7%)
99.2	1 (16.7%)
99.3	1 (16.7%)
99.4	1 (16.7%)
99.5	1 (16.7%)
99.6	1 (16.7%)
99.7	1 (16.7%)
99.9	1 (16.7%)
100.0	
Any violation of max point dose [a]	
No	6 (100.0%)

[a] Rows are not mutually exclusive; patients may belong to more than one category

Abbreviations: PTV, planning target volume; Gy, Gray.

Supplementary Table 4. Grade 2 or higher adverse events regardless of attribution

**Event**	**N (%) patients with event**		
	**Grade ≥ 2**	**Grade 2**	**Grade 3**
Any Event	5 (83.3%)	3 (50.0%)	2 (33.3%)
Fatigue	3 (50.0%)	2 (33.3%)	1 (16.7%)
Cough	2 (33.3%)	2 (33.3%)	0 (0.0%)
Alkaline phosphatase increased	1 (16.7%)	1 (16.7%)	0 (0.0%)
Anorexia	1 (16.7%)	1 (16.7%)	0 (0.0%)
Ataxia	1 (16.7%)	0 (0.0%)	1 (16.7%)
Cognitive disturbance	1 (16.7%)	1 (16.7%)	0 (0.0%)
Constipation	1 (16.7%)	1 (16.7%)	0 (0.0%)
Cushingoid	1 (16.7%)	1 (16.7%)	0 (0.0%)
Dyspnea	1 (16.7%)	0 (0.0%)	1 (16.7%)
Generalized muscle weakness	1 (16.7%)	1 (16.7%)	0 (0.0%)
Headache	1 (16.7%)	1 (16.7%)	0 (0.0%)
Myalgia	1 (16.7%)	1 (16.7%)	0 (0.0%)
Pleural effusion	1 (16.7%)	0 (0.0%)	1 (16.7%)
Seizure	1 (16.7%)	1 (16.7%)	0 (0.0%)
Urinary tract infection	1 (16.7%)	1 (16.7%)	0 (0.0%)
Weight loss	1 (16.7%)	1 (16.7%)	0 (0.0%)

Supplementary Table 5. Number of patients evaluated for patient-reported outcomes (EQ-5D-5L, MDASI) and investigator-assessed neurological evaluation (NANO

Instrument	N (%)				
	**Baseline**	**Cycle 1**	**Cycle 2**	**Subsequent Cycles**	**EOT**
EQ-5D-5L	1 (16.7%)	2 (33.3%)	0 (0.0%)	1 (16.7%)	2 (33.3%)
NANO Scale	4 (66.7%)	3 (50.0%)	4 (66.7%)	1 (16.7%)	3 (50.0%)
MDASI	1 (16.7%)	2 (33.3%)	0 (0.0%)	1 (16.7%)	2 (33.3%)

Abbreviations: EOT, end of treatment; EQ-5D-5L, EuroQol 5-dimension 5-level questionnaire; NANO, Neurologic Assessment in Neuro-Oncology; MDASI, MD Anderson Symptom Inventory.

Supplementary Fig. 1
NANO Scale Scores over time in Case Number 1 (on treatment for ~54 weeks)

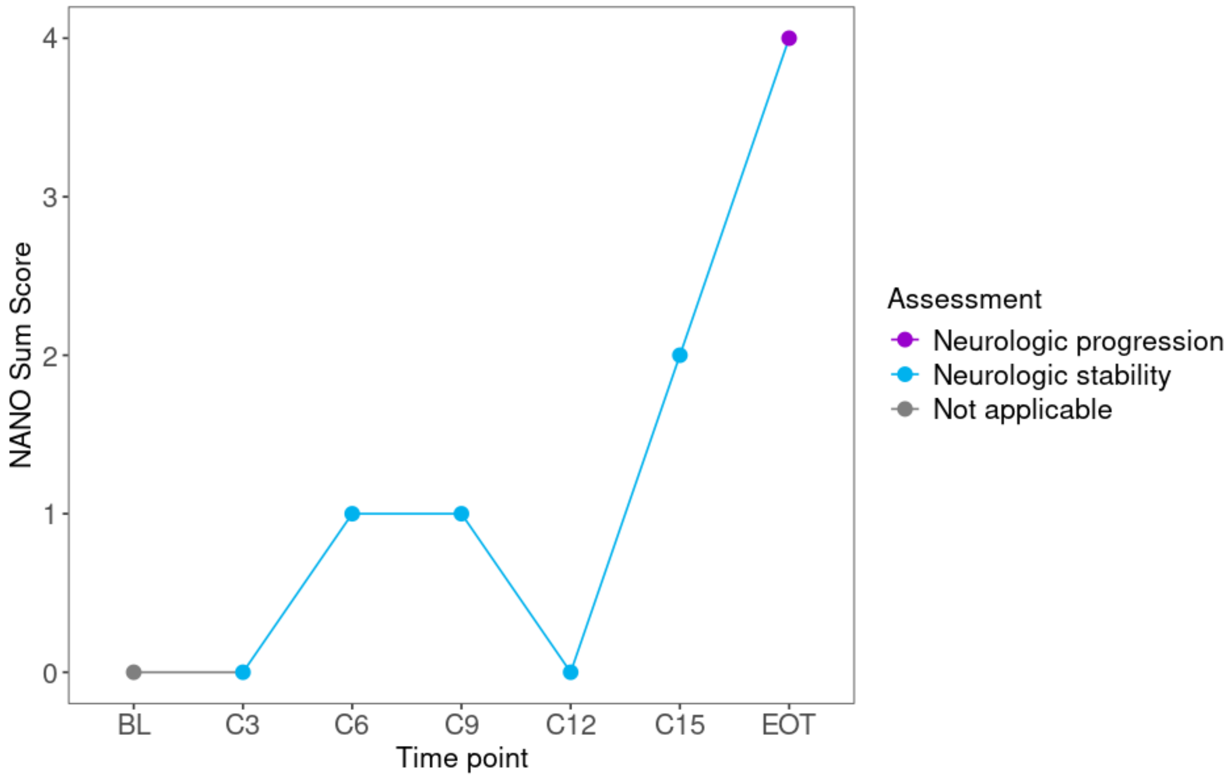

Abbreviations: NANO, Neurologic Assessment in Neuro-Oncology.

Supplementary Fig. 2
EQ-5D-5L Scores over time for Case Number 1 (on treatment for ~54 weeks)

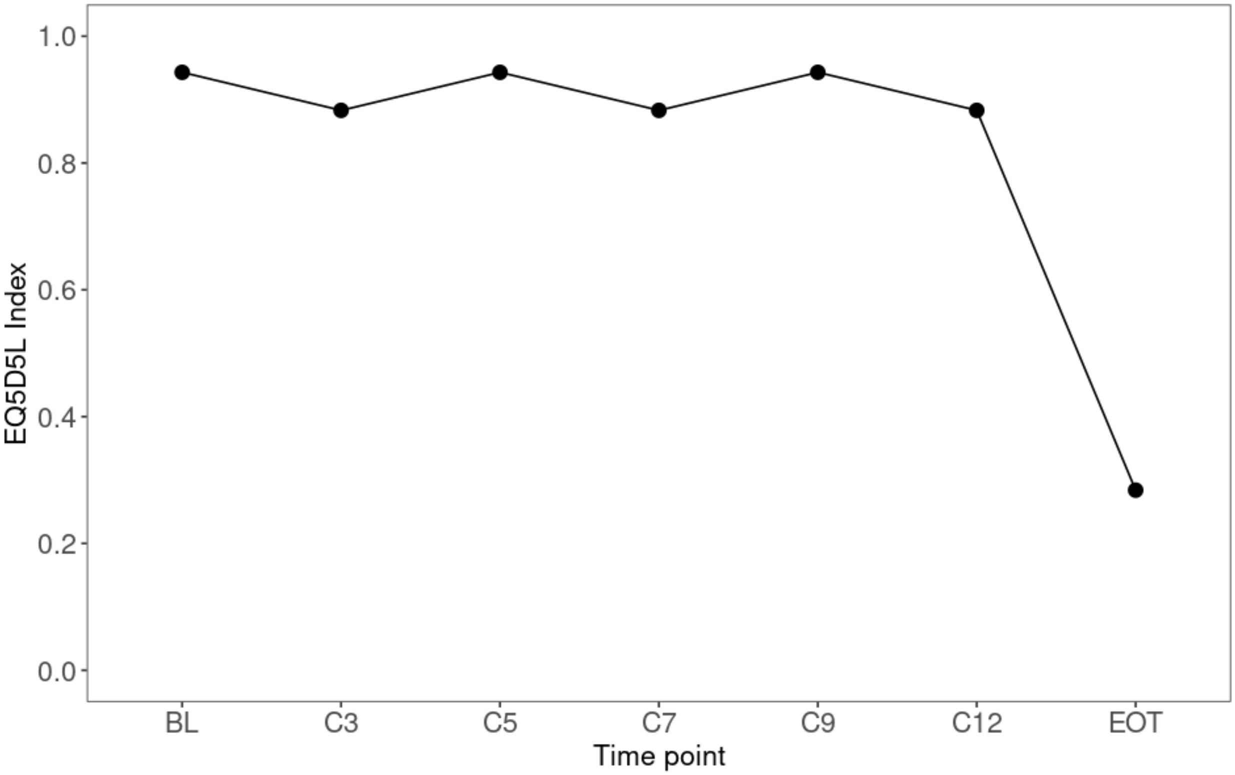

Note: Lower scores indicate poorer self-reported health status.

Abbreviations: EQ-5D-5L, EuroQol 5-dimension 5-level questionnaire.

Supplementary Fig. 3
MDASI symptom scores over time for Case Number 1 (on treatment for ~54 weeks)

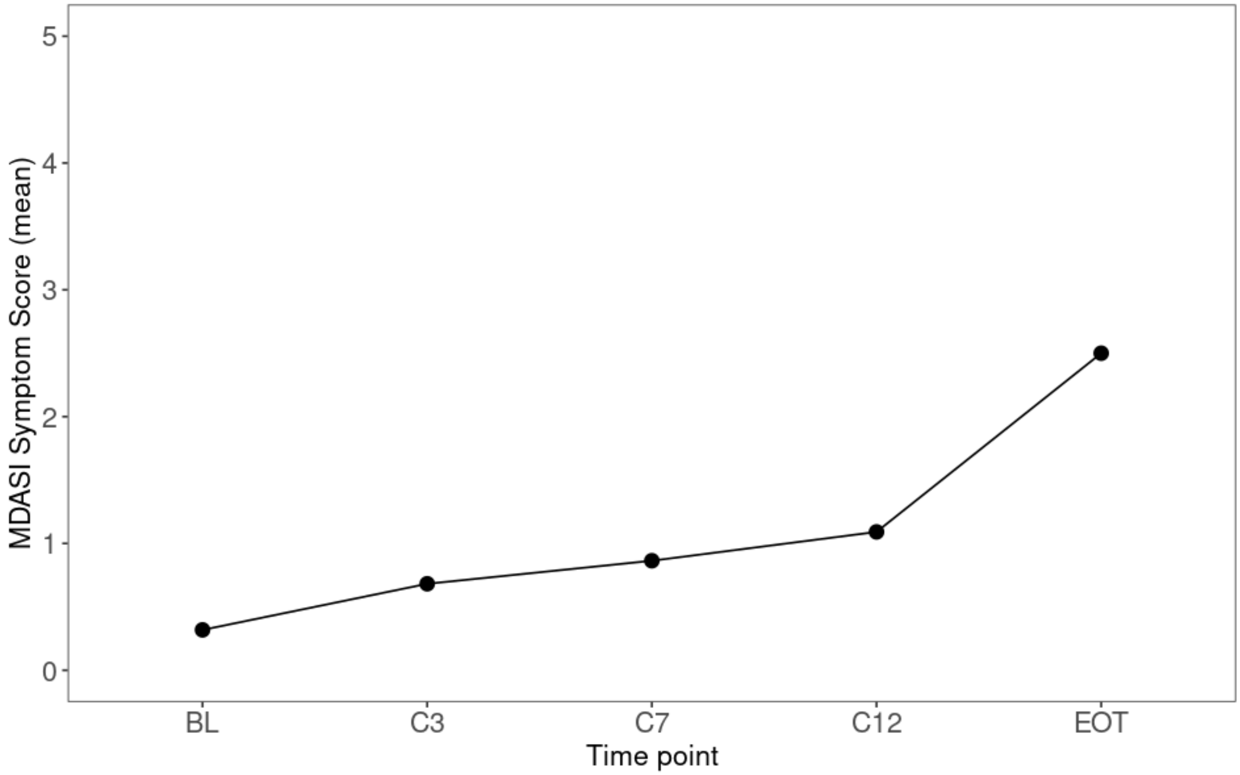

Note: 22 questions are posed, with a score of 0-10. A higher score means greater symptom severity.

Abbreviations: MDASI, MD Anderson Symptom Inventory.

Supplementary Fig. 4
MDASI interference scores over time for Case Number 1 (on treatment for ~54 weeks)

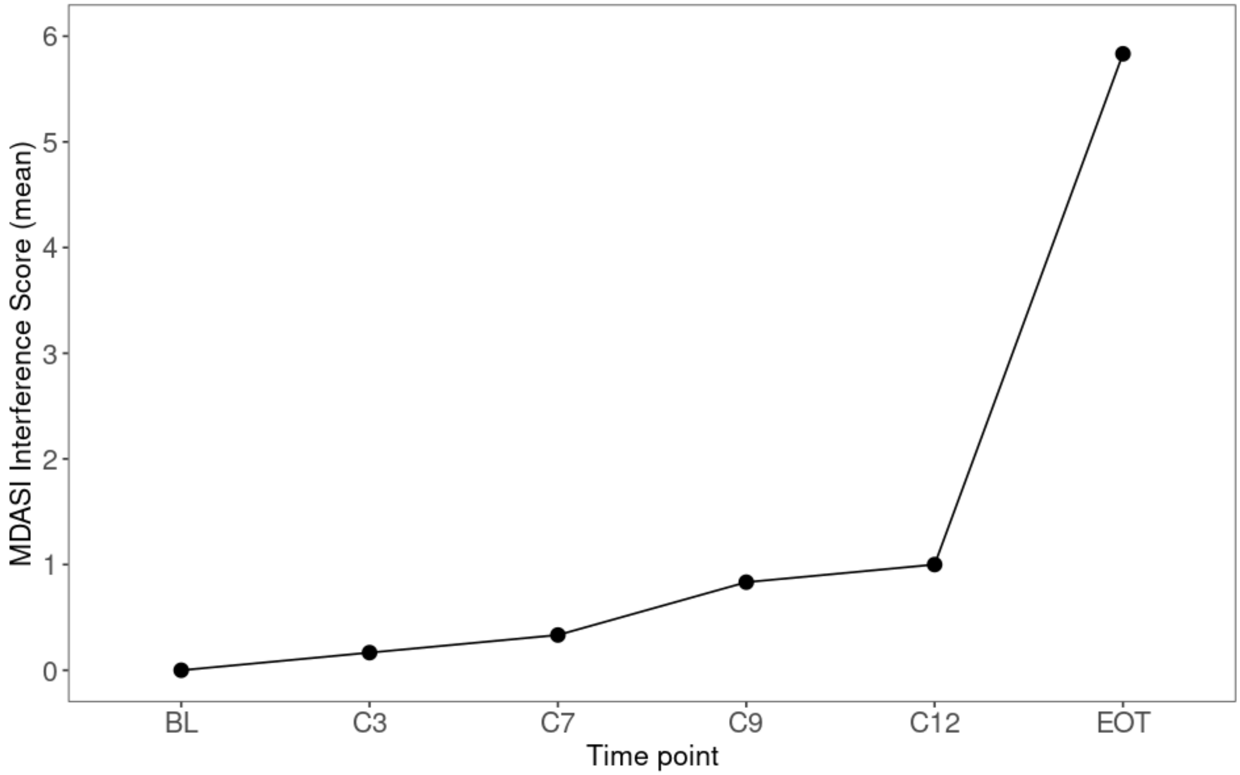

Note: Six questions are posed with a score of 0-10. A higher score means more interference of symptoms on daily life.

Abbreviations: MDASI, MD Anderson Symptom Inventory.
